# Synergistic and additive interactions of *Shewanella* sp., *Pseudomonas* sp*.* and *Thauera* sp. with chlorantraniliprole and emamectin benzoate for controlling *Spodoptera litura* (Fabricius)

**DOI:** 10.1038/s41598-023-41641-0

**Published:** 2023-09-05

**Authors:** Sunaina Sarkhandia, Geetika Sharma, Rohit Mahajan, Satish Koundal, Manoj Kumar, Pooja Chadha, Harvinder Singh Saini, Sanehdeep Kaur

**Affiliations:** 1 Department of Zoology, Guru Nanak Dev University, Amritsar, Punjab 143005 India; 2 Department of Microbiology, Guru Nanak Dev University, Amritsar, Punjab 143005 India

**Keywords:** Zoology, Entomology, Environmental sciences, Environmental impact

## Abstract

The imprudent use of insecticides causes the development of resistance in insect pest populations, contamination of the environment, biological imbalance and human intoxication. The use of microbial pathogens combined with insecticides has been proposed as an alternative strategy for insect pest management. This IPM approach may offer effective ways to control pests, in addition to lowering the risk of chemical residues in the environment. *Spodoptera litura* (Fabricius) is a major pest of many crops like cotton, maize, tobacco, cauliflower, cabbage, and fodder crops globally. Here, we evaluated the combined effects of new chemistry insecticides (chlorantraniliprole and emamectin benzoate) and entomopathogenic bacterial strains, *Shewanella* sp. (SS4), *Thauera *sp. (M9) and *Pseudomonas* sp. (EN4) against *S. litura* larvae inducing additive and synergistic interactions under laboratory conditions. Both insecticides produced higher larval mortality when applied in combination with bacterial isolates having maximum mortality of 98 and 96% with LC_50_ of chlorantraniliprole and emamectin benzoate in combination with LC_50_ of *Pseudomonas* sp. (EN4) respectively. The lower concentration (LC_20_) of both insecticides also induced synergism when combined with the above bacterial isolates providing a valuable approach for the management of insect pests. The genotoxic effect of both the insecticides was also evaluated by conducting comet assays. The insecticide treatments induced significant DNA damage in larval hemocytes that further increased in combination treatments. Our results indicated that combined treatments could be a successful approach for managing *S*. *litura* while reducing the inappropriate overuse of insecticides.

## Introduction

The tobacco cutworm, *Spodoptera litura* (Fabricius) (Lepidoptera: Noctuidae), is a notorious polyphagous pest of many field crops, including cotton, corn, groundnut, soybean, tobacco, and vegetables^1^. It is found throughout temperate and tropical Asia, Australasia, and Pacific Islands^2^. Early instar larvae are gregarious feeders while the later instars disperse and feed voraciously, causing complete defoliation of plants when present in abundance^3^. A variety of insecticides with different mechanisms of action are being used to manage *S. litura*. However, there are reports indicating the development of resistance in *S. litura* to many of the commonly used insecticides^4,5^. Although chemical insecticides are the most reliable tool in insect pest management but resistance to insecticides is a major problem associated with the chemical control of insect pests. Imprudent spraying and repeated use of insecticides including chlorinated hydrocarbons, carbamates, organophosphates, and pyrethroids not only led to the development of resistance in many insect pests but also posed a threat to beneficial creatures such as crop insect natural enemies, pollinators, and non-target biodiversity as well as environment^6,7^. Therefore, to overcome these problems and to minimize insecticide applications it becomes imperative to look for alternative methods of pest management.

Among the alternatives, microbial control agents including bacteria, fungi, viruses and nematodes are getting serious attention due to their environmental safety and pest selectivity. Entomopathogenic bacteria are the most effective microbes for pest management, with *Bacillus thuringiensis* (*Bt*) dominating the market having 2% of the insecticidal market share^8^. Besides *Bt*, *Serratia*, *Pseudomonas*, *Xenorhabdus, Pseudomonas cedrina*, *Paenibacillus* spp., *Lysinibacillus sphaericus*, and *Chromobacterium substugae* have also been reported for insecticidal activity against coleopteran, lepidopteran, and dipteran pests under field conditions^9–12^. Biocontrol agents have a number of advantages including their target specificity, reduced potential for development of resistance in target pest, and safety to non-target species, however, chemical insecticides still predominate as the primary control strategy in most systems. In general the major limitations in adoption of microbial control are, an extended time to cause sufficient larval mortality relative to chemical insecticide, narrow host range, cost of production and susceptibility to environmental degradation. Microbial control agents have been found to be very effective when combined with lower concentrations of insecticides^13^. When two control agents work independently on the same target host and do not affect the toxicity of one another, their combined effects can be additive or synergistic^14^. Combining multiple control agents can improve the efficacy of IPM techniques and provide a cost-effective and time-saving alternative for pest management. Moreover, synthetic insecticides cannot currently be eliminated but their use can be reduced by utilizing them in conjunction with entomopathogenic microorganisms^15^. New chemistry insecticides with novel modes of action, like spinosad, indoxacarb, abamectin, emamectin benzoate, chlorantraniliprole, lufenuron and fipronil, have now been introduced for the management of many insect pests. These compounds are generally species-specific and less harmful^16–18^. To achieve pollution-free agricultural output and good compatibility with the environment, we may utilize new chemical pesticides at lower doses coupled with entomopathogenic bacterial strains. Combined treatments of insecticide and biological agents can be more efficient than individual constituents because of their different modes of action, which may also delay the development of resistance^19^. Entomopathogenic bacteria appear to be compatible with a wide spectrum of chemical insecticides and can result in synergism when applied in combination even at low pesticide doses^20–22^.

Considering the significance of combination approaches, the present study was designed to evaluate the compatibility of bacterial strains with insecticides. Three bacterial isolates viz. *Shewanella* sp. (SS4), *Thauera *sp. (M9) and *Pseudomonas* sp*.* (EN4) showing pathogenicity against *S. litura* were used for combination treatments with the two new chemistry insecticides i.e., chlorantraniliprole (Cg) and emamectin benzoate (Eb). To determine if the combined effects were antagonistic, additive, or synergistic, we compared the combination treatments with individual bacterial and insecticide treatments. Additionally, we examined the genotoxicity of insecticides and bacterial strains on *S. litura*.

## Results

### Estimation of sub-lethal concentrations of insecticides

**Figure 1:**
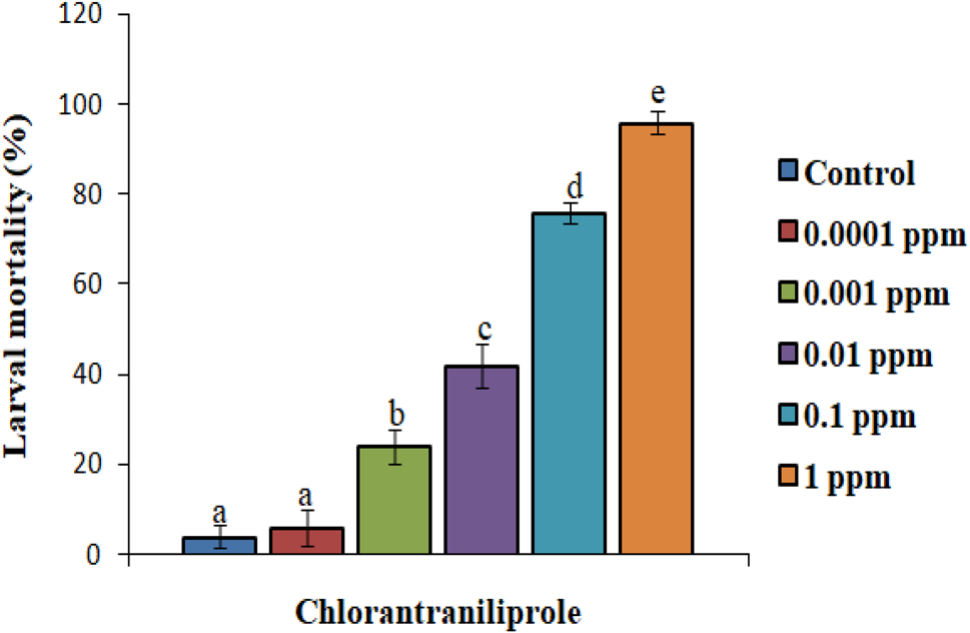
Screening of different concentrations of chlorantraniliprole for insecticidal potential against second instar *S. litura* larvae. Bars represent the Mean ± SE. Different letters above the bars represent significant differences at Tukey’s test p ≤ 0.05.

**Figure 2:**
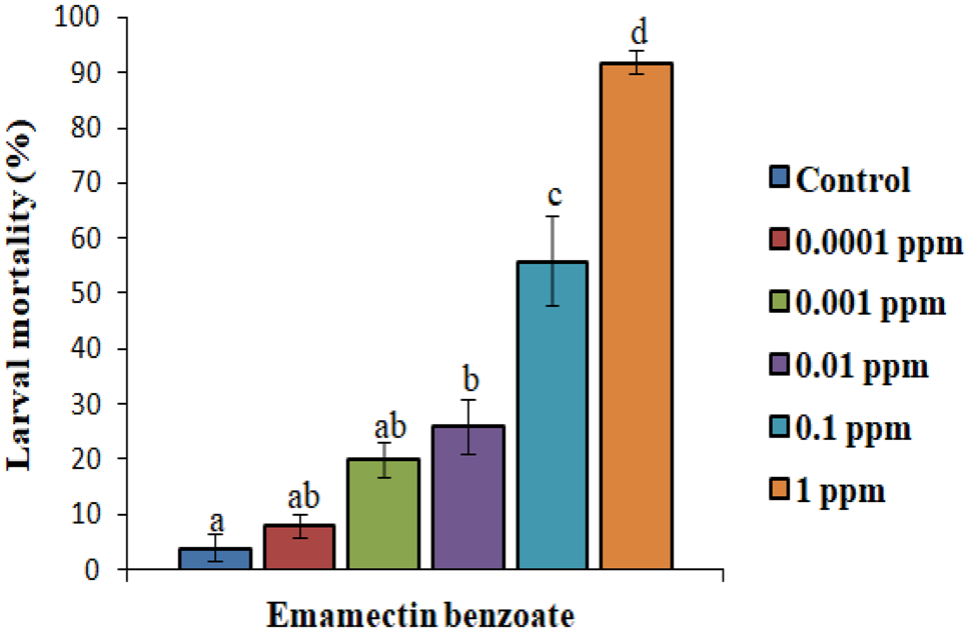
Screening of different concentrations of emamectin benzoate for insecticidal potential against second instar *S. litura* larvae. Bars represent the Mean ± SE. Different letters above the bars represent significant differences at Tukey’s test p ≤ 0.05.

**Figure 3:**
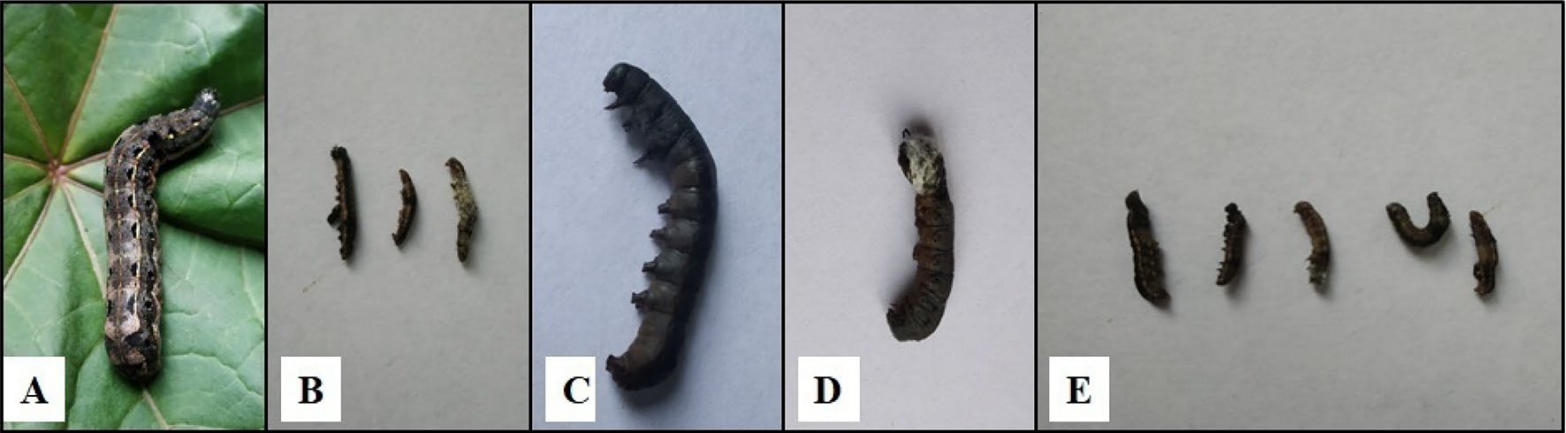
Larval mortality due to treatment with insecticides and bacterial suspensions (**A**) Healthy larva, (**B**) Dead larvae due to insecticide treatments, (**C**,**D**) Dead larva due to bacterial infection and (**E**) Dead larvae due to combined treatments.

In case of chlorantraniliprole, except for the lower concentration, all the concentrations induced significantly higher larval mortality over control. The mortality rate increased significantly from 24 to 96% in a dose-dependent manner (F_(5, 24)_ = 115.67, p ≤ 0.05) (Fig. 1). The lower lethal and median lethal values of chlorantraniliprole against *S. litura* larvae after 72 h of treatment were found to be: LC_20_ = 0.001 ppm (95% confidence interval 0.001–0.002 ppm) and LC_50_ = 0.011 ppm (95% confidence interval 0.008–0.017 ppm). Similarly, with respect to control, all the concentrations of emamectin benzoate significantly increased the larval mortality except for the lower two concentrations (F_(5, 24)_ = 58.79, p ≤ 0.05) (Fig. 2). The LC_20_ and LC_50_ value of emamectin benzoate against *S. litura* were 0.002 ppm (95% confidence interval: 0.000 – 0.009 ppm) and 0.032 ppm (95% confidence interval: 0.006 – 0.368 ppm) respectively after 72 h of treatment. Insecticide treatment reduced feeding in larvae, induced paralysis that ultimately led to larval death while the larvae of control group were found to be healthy (Fig. 3A,B).

### Compatibility between insecticide and bacterial cultures

**Figure 4:**
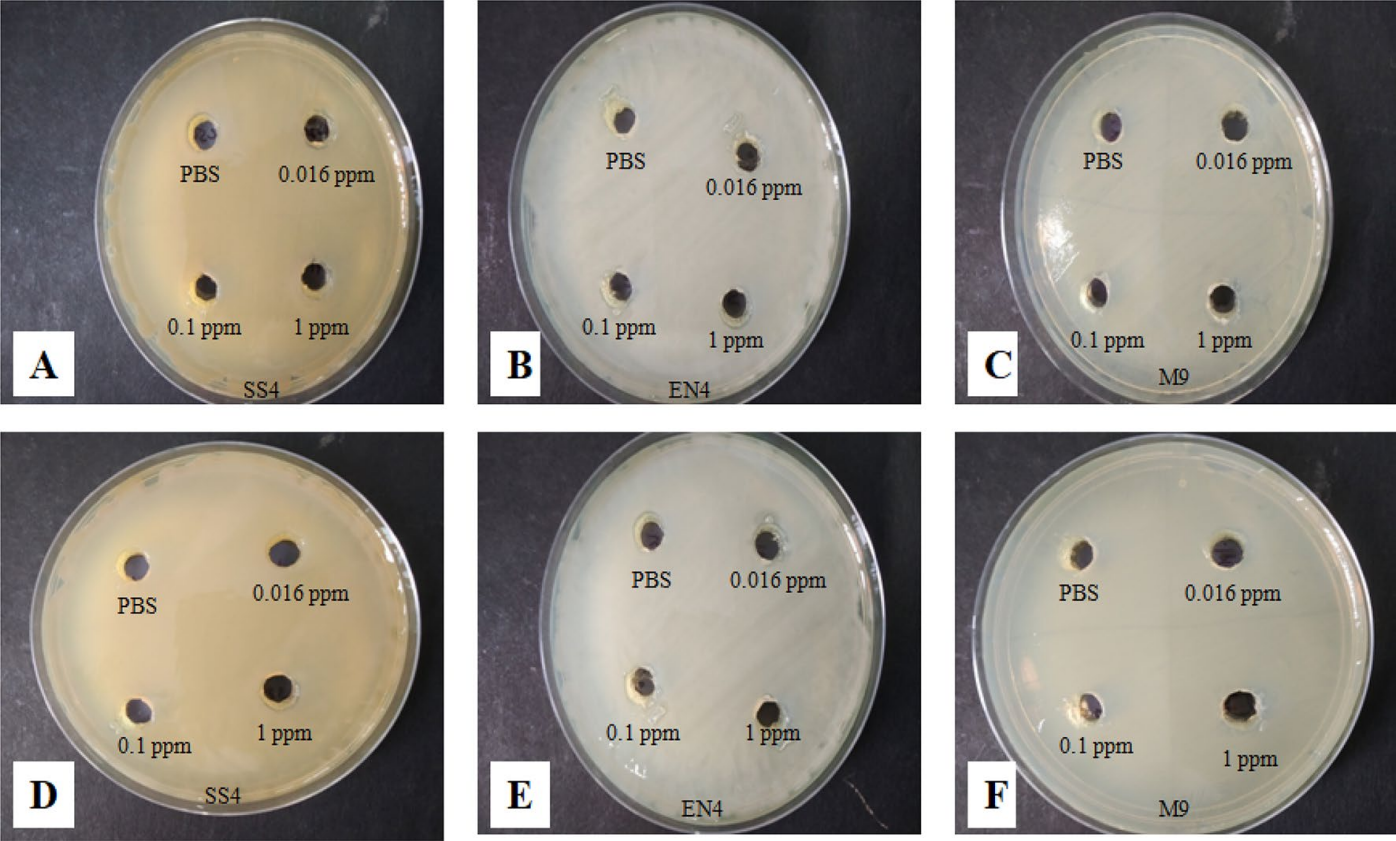
LB plates indicating the compatibility between insecticides and bacterial cultures. (**A**–**C**) Compatibility between chlorantraniliprole and bacterial cultures (SS4, EN4 and M9) and (**D**–**F**) Compatibility between emamectin benzoate and bacterial cultures (SS4, EN4 and M9).

The shake flask assay indicated no difference in the growth of bacterial cells in flask containing different concentrations of the insecticide (chlorantraniliprole and emamectin benzoate) and the control flask. Similarly, in the plate assay bacterial growth around the wells containing different concentrations of the insecticides was same as in case of control well containing PBS only. There were no clear zones in the plates, indicating the compatibility between both the insecticides and bacterial cultures (Fig. 4).

### Effect of different concentrations of insecticide and bacteria against *S. litura*

As is evident from results presented in Tables 1 and 2, insecticidal and bacterial treatments did not differ significantly in terms of larval mortality in *S. litura*. The LC_50_ concentrations of SS4, EN4 and M9 caused 46, 48 and 42% of larval mortality respectively after treatment. *S. litura* larvae treated with bacterial cell suspension stopped feeding, turned black and eventually died (Fig. 3C,D). The larvae fed on LC_20_ and LC_50_ concentrations of chlorantraniliprole suffered 24 and 50% of larval mortality while LC_20_ and LC_50_ concentrations of emamectin benzoate induced 26 and 52% of larval mortality.

**Table 1:** Effect of combined treatments of chlorantraniliprole (Cg) and bacterial cell suspensions [*Shewanella* sp. (SS4), *Pseudomonas* sp. (EN4) and *Thauera* sp. (M9)] on cumulative larval mortality of *S. litura*.

Number of experiments	Treatments	Observed mortality	Expected mortality	χ^2^ value	Interaction
Individual treatments for experiment 1 and 2	LC_50_ Cg	50.0 ± 7.74^a^	–	–	–
	LC_50_ SS4	46.0 ± 2.44^a^	–	–	–
	LC_50_ EN4	48.0 ± 5.83^a^	–	–	–
	LC_50_ M9	42.0 ± 7.34^a^	–	–	–
Combination treatments for experiment 1	LC_50_ Cg + LC_50_ SS4	80.0 ± 3.16^b^	73.0	0.67	Additive
	LC_50_ Cg + LC_50_ EN4	88.0 ± 3.74^b^	74.0	2.64	Additive
	LC_50_ Cg + LC_50_ M9	78.0 ± 2.00^b^	71.0	0.69	Additive
F_(6, 28)_ – value		14.43**	–	–	–
Combination treatments for experiment 2	LC_50_ Cg + LC_50_ SS4	90.0 ± 3.16^b^	73.0	3.95	Synergistic
	LC_50_ Cg + LC_50_ EN4	92.0 ± 3.74^b^	74.0	4.37	Synergistic
	LC_50_ Cg + LC_50_ M9	86.0 ± 5.09^b^	71.0	3.16	Additive
F_(6, 28)_ – value		18.29**	–	–	–
Individual treatments for experiment 3	LC_20_ Cg	24.0 ± 5.09^a^	–	–	–
	LC_50_ Cg	50.0 ± 7.74^ab^	–	–	–
	LC_50_ SS4	46.0 ± 2.44^ab^	–	–	–
	LC_50_ EN4	48.0 ± 5.83^ab^	–	–	–
	LC_50_ M9	42.0 ± 7.34^a^	–	–	–
Combination treatments for experiment 3	LC_20_ Cg + LC_50_ SS4	80.0 ± 6.32^c^	58.96	7.50	Synergistic
	LC_20_ Cg + LC_50_ EN4	84.0 ± 6.78^c^	60.48	9.14	Synergistic
	LC_20_ Cg + LC_50_ M9	74.0 ± 10.29^bc^	55.92	5.84	Synergistic
	LC_50_ Cg + LC_50_ SS4	94.0 ± 4.0^c^	73.0	6.04	Synergistic
	LC_50_ Cg + LC_50_ EN4	98.0 ± 2.0^c^	74.0	7.78	Synergistic
	LC_50_ Cg + LC_50_ M9	92.0 ± 4.89^c^	71.0	6.21	Synergistic
F_(10, 44)_ – value		16.88**	–	–	–

Number of insecticide treatments in combination with bacterial treatments in Combined Experiment 1 = 1, Combined Experiment 2 = 3 and Combined Experiment 3 = 6. Figures are Mean ± Standard Error for observed mortality. Means followed by different superscript letters within a column are significantly different. Tukey’s test, p ≤ 0.05, **Significant at 1%^64^. Expected mortality ME = MB + MI (1 − MB), where MB and MI are the observed mortality percentage caused by bacteria and insecticide alone. Test for interaction based on χ^2^ with 1 df, using the formula χ^2^ = (MBI − ME)^2^/ME, wherein MBI is the observed mortality caused by bacteria + insecticide.

**Table 2:** Effect of combined treatments of emamectin benzoate (Eb) and bacterial cell suspensions [*Shewanella* sp. (SS4), *Pseudomonas* sp. (EN4) and *Thauera* sp. (M9)] on cumulative larval mortality of *S. litura*.

Number of experiments	Treatments	Observed mortality	Expected mortality	χ^2^ value	Interaction
Individual treatments for experiment 1 and 2	LC_50_ Eb	52.0 ± 3.74^a^	–	–	–
	LC_50_ SS4	46.0 ± 2.44^a^	–	–	–
	LC_50_ EN4	48.0 ± 5.83^a^	–	–	–
	LC_50_ M9	42.0 ± 7.34^a^	–	–	–
Combination treatments for experiment 1	LC_50_ Eb + LC_50_ SS4	82.0 ± 4.89^b^	74.08	0.84	Additive
	LC_50_ Eb + LC_50_ EN4	86.0 ± 4.00^b^	75.04	1.60	Additive
	LC_50_ Eb + LC_50_ M9	78.0 ± 2.00^b^	72.16	0.47	Additive
F_(6, 28)_ – value		16.76**	–	–	–
Combination treatments for experiment 2	LC_50_ Eb + LC_50_ SS4	88.0 ± 3.74^b^	74.08	2.61	Additive
	LC_50_ Eb + LC_50_ EN4	94.0 ± 2.44^b^	75.04	4.79	Synergistic
	LC_50_ Eb + LC_50_ M9	84.0 ± 5.09^b^	72.16	1.94	Additive
F_(6,28)_ – value		23.32**	–	–	–
Individual treatments for experiment 3	LC_20_ Eb	26.0 ± 2.44^a^	–	–	–
	LC_50_ Eb	52.0 ± 3.74^bc^	–	–	–
	LC_50_ SS4	46.0 ± 2.44^b^	–	–	–
	LC_50_ EN4	48.0 ± 5.83^b^	–	–	–
	LC_50_ M9	42.0 ± 7.34^ab^	–	–	–
Combination treatments for experiment 3	LC_20_ Eb + LC_50_ SS4	74.0 ± 4.0^de^	60.04	3.24	Additive
	LC_20_ Eb + LC_50_ EN4	78.0 ± 3.74^def^	61.52	4.41	Synergistic
	LC_20_ Eb + LC_50_ M9	70.0 ± 4.47^cd^	57.08	2.92	Additive
	LC_50_ Eb + LC_50_ SS4	92.0 ± 2.0^ef^	74.08	4.33	Synergistic
	LC_50_ Eb + LC_50_ EN4	96.0 ± 2.44^f^	75.04	5.85	Synergistic
	LC_50_ Eb + LC_50_ M9	90.0 ± 3.16^ef^	72.16	4.41	Synergistic
F_(10, 44)_—value		32.63**	–	–	–

Number of insecticide treatments in combination with bacterial treatments in Combined Experiment 1 = 1, Combined Experiment 2 = 3 and Combined Experiment 3 = 6. Figures are Mean ± Standard Error for observed mortality. Means followed by different superscript letters within a column are significantly different. Tukey’s test, p ≤ 0.05, **Significant at 1%^64^. Expected mortality ME = MB + MI (1 − MB), where MB and MI are the observed mortality percentage caused by bacteria and insecticide alone. Test for interaction based on χ^2^ with 1 df, using the formula χ^2^ = (MBI − ME)^2^/ME, wherein MBI is the observed mortality caused by bacteria + insecticide.

### Combined effects of bacteria and insecticide against *S. litura*

The studies revealed significant effect on larval mortality due to combined treatments when compared with individual treatments (Fig. 3E). The combination treatment of chlorantraniliprole with bacterial isolates i.e. SS4, M9 and EN4 induced higher mortality (78–88%) in *S litura* larvae used in the first experiment (F_(6, 28)_ = 14.43, p ≤ 0.05). Additive interaction was found in all the combinations (χ^2^ ˂ 3.84) (Table 1). In the second experiment, all the combination treatments increased the larval mortality in a synergistic way (F_(6, 28)_ = 18.29, p ≤ 0.05) (χ^2^ > 3.84) except for M9 culture (LC_50_ Cg + LC_50_ M9). When alternate insecticide and bacterial treatments were given to larvae in the third experiment, the mortality rate increased further. All the combination treatments exhibited synergistic impact indicating that each bacterial isolate and insecticide contributed to *S. litura* larval mortality (Table 1). The combined treatment, LC_50_ Cg + LC_50_ EN4 caused maximum larval mortality (98%) (χ^2^ > 3.84) compared to other groups (F_(10, 44)_ = 16.88, p ≤ 0.05). The lower concentration (LC_20_) of chlorantraniliprole when combined with bacteria also induced synergistic effect thus providing a valuable approach for the control of insect pests.

As for emamectin benzoate (Table 2), we observed an additive effect in all combinations of first experiment (χ^2^ < 3.84; p ≤ 0.05). The results of second experiment indicated synergistic effect in the combination treatment, LC_50_ Eb + LC_50_ EN4 with χ^2^ value of 4.79 (F_(6, 28)_ = 23.32, p ≤ 0.05) while additive effects were recorded in other treatments (Table 2). Synergistic effect was observed across all the combination treatments in the third experiment except for LC_20_ Eb + LC_50_ SS4 and LC_20_ Eb + LC_50_ M9 treatments that exhibited additive effect (F_(10, 44)_ = 32.63, p ≤ 0.05). Overall, these results indicated that combining insecticide with bacterial treatment increased the mortality rate of *S. litura* larvae.

### Comet assay

**Figure 5:**
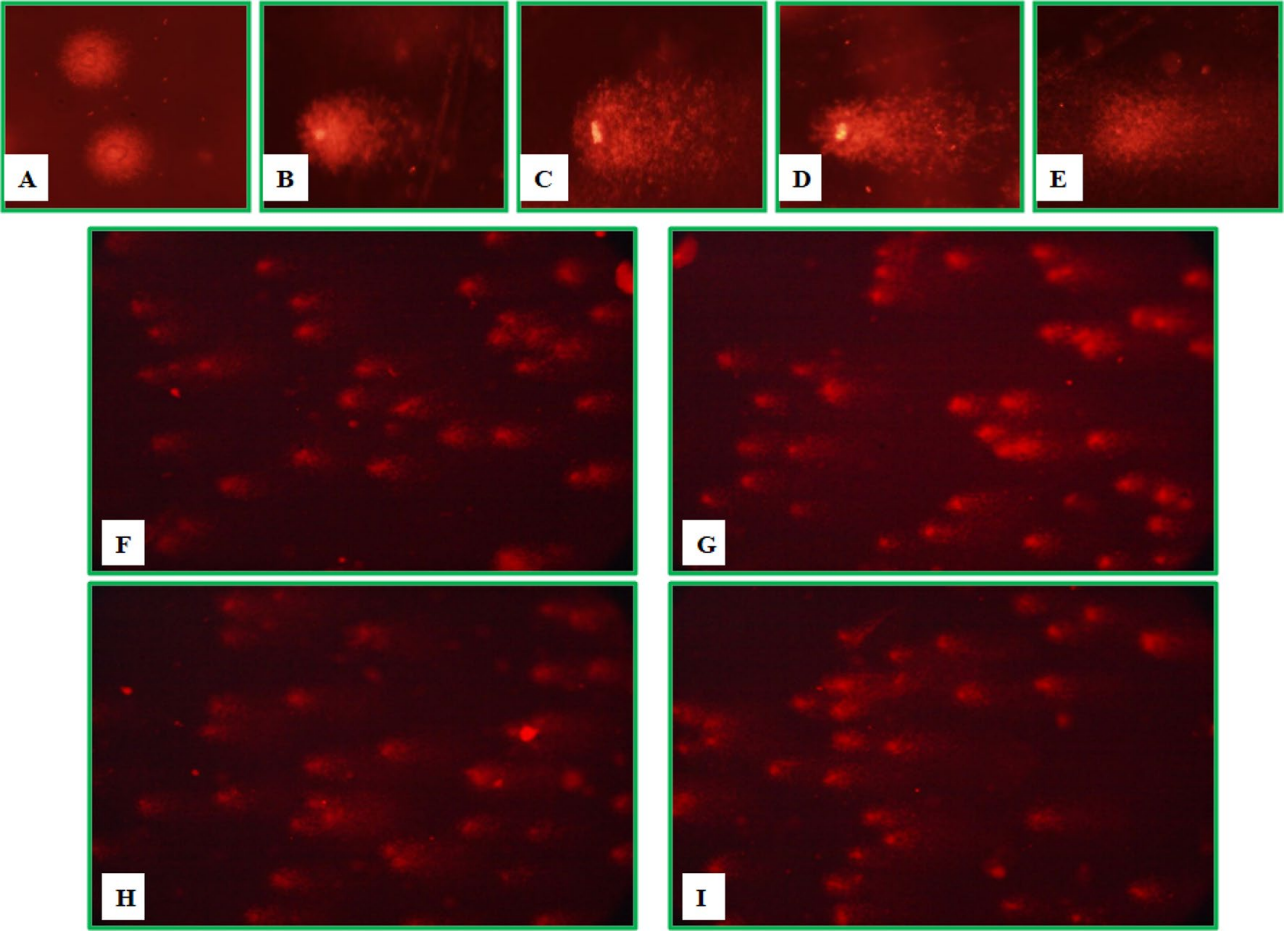
DNA damage in hemocytes of *S. litura* (**A**) Control; Individual treatments: (**B**) LC_20_ chlorantraniliprole (Cg), (**C**) LC_20_ emamectin benzoate (Eb), (**D**) LC_50_ chlorantraniliprole, (**E**) LC_50_ emamectin benzoate, Combined treatments: (**F**) LC_20_ chlorantraniliprole and bacterial cell suspensions, (**G**) LC_50_ chlorantraniliprole and bacterial cell suspensions, (**H**) LC_20_ emamectin benzoate and bacterial cell suspensions and (**I**) LC_50_ emamectin benzoate and bacterial cell suspensions.

In the current study, treatment with chlorantraniliprole and emamectin benzoate induced genotoxic effects in *S. litura* larvae. The larvae treated with insecticide and bacterial cell suspension i.e. combination treatments, had much higher levels of damage in larval hemocytes as compared to control and individual treatments (Fig. 5).

The tail length was significantly increased in individual treatments of both the insecticides as compared to control, however, the increase in tail length was observed to be higher in combination treatment groups as compared to individual exposure groups. In case of chlorantraniliprole, it significantly increased from 10.73 μm in control (distilled water) to 12.76 μm and 16.63 μm in LC_20_ and LC_50_ concentrations, respectively. However, the tail length further increased in combination treatments with a maximum value of 35.69 μm in LC_50_ Cg + LC_50_ EN4 (F_(9, 20)_ = 277.06, p ≤ 0.05). A similar pattern was also observed for tail length in larvae treated with emamectin benzoate with maximum increase of 43.73 μm in LC_50_ Eb + LC_50_ EN4 treatment (F_(9, 20)_ = 572.04, p ≤ 0.05) (Table 3). With respect to control, the percent tail DNA values also increased significantly in both the individual insecticide (LC_20_ and LC_50_) and the combination treatment groups. Likewise, TM and OTM values were analyzed to be significantly and maximally increased in combined treatment groups of chlorantraniliprole and emamectin benzoate in comparison to their individual and control groups (Tables 3, 4).

**Table 3:** Effect of individual chlorantraniliprole (Cg) treatments and its combinations with bacterial cell suspensions [*Shewanella* sp. (SS4), *Pseudomonas* sp. (EN4) and *Thauera* sp. (M9)] on genotoxic parameters of *S. litura* after 96 h of treatment.

Treatments	Tail length	% Tail DNA	Tail Moment	Olive tail moment
Control (distilled H_2_O)	10.73 ± 0.12^ab^	3.14 ± 0.02^a^	0.53 ± 0.01^a^	1.55 ± 0.02^a^
Control (PBS)	9.94 ± 0.28^a^	3.91 ± 0.21^a^	0.64 ± 0.02^a^	1.80 ± 0.10^a^
LC_20_ Cg	12.76 ± 0.13^b^	16.63 ± 0.32^b^	1.36 ± 0.18^a^	1.89 ± 0.15^a^
LC_50_ Cg	16.63 ± 0.52^c^	20.33 ± 0.44^c^	2.10 ± 0.05^a^	2.26 ± 0.14^a^
LC_20_ Cg + LC_50_ SS4	26.72 ± 0.63^e^	23.03 ± 0.43^d^	6.70 ± 0.02^b^	8.71 ± 0.18^c^
LC_20_ Cg + LC_50_ EN4	31.32 ± 0.76^f^	35.0 ± 0.45^f^	12.04 ± 0.84^ cd^	15.90 ± 0.33^d^
LC_20_ Cg + LC_50_ M9	21.80 ± 0.20^d^	23.66 ± 0.69^de^	5.25 ± 0.42^b^	6.81 ± 0.18^b^
LC_50_ Cg + LC_50_ SS4	29.64 ± 0.56f.	35.70 ± 0.11f.	13.09 ± 0.07^d^	17.74 ± 0.48^e^
LC_50_ Cg + LC_50_ EN4	35.69 ± 1.05^g^	43.60 ± 0.66^g^	16.66 ± 0.52^e^	20.96 ± 0.42^f^
LC_50_ Cg + LC_50_ M9	23.29 ± 0.34^d^	25.70 ± 0.11^e^	10.87 ± 0.18^c^	14.80 ± 0.12^d^
F_(9, 20)_—value	277.06**	1009.0**	275.98**	850.60**

Figures are Mean ± Standard Error. Means followed by different superscript letters within a column are significantly different. Tukey’s test, p ≤ 0.05, **Significant at 1%

**Table 4:** Effect of individual emamectin benzoate (Eb) treatments and its combination with bacterial cell suspensions [*Shewanella* sp. (SS4), *Pseudomonas* sp. (EN4) and *Thauera* sp. (M9)] on genotoxic parameters of *S. litura* after 96 h of treatment.

Treatments	Tail length	% Tail DNA	Tail moment	Olive tail moment
Control (Distlled H_2_O)	10.73 ± 0.12^ab^	3.14 ± 0.02^a^	0.53 ± 0.01^a^	1.55 ± 0.02^a^
Control (PBS)	9.94 ± 0.28^a^	3.91 ± 0.21^a^	0.64 ± 0.02^a^	1.80 ± 0.10^a^
LC_20_ Eb	13.73 ± 0.46^b^	6.74 ± 0.13^b^	1.06 ± 0.08^a^	6.90 ± 0.45^b^
LC_50_ Eb	16.46 ± 0.29^c^	9.16 ± 0.31^c^	1.70 ± 0.11^ab^	8.53 ± 0.17^c^
LC_20_ Eb + LC_50_ SS4	17.96 ± 1.16^c^	12.68 ± 0.10^d^	2.63 ± 0.23^b^	11.41 ± 0.19^d^
LC_20_ Eb + LC_50_ EN4	28.03 ± 0.23^e^	21.50 ± 0.04^f^	16.85 ± 0.08^d^	14.16 ± 0.08^fg^
LC_20_ Eb + LC_50_ M9	22.53 ± 0.06^d^	15.73 ± 0.07^e^	7.23 ± 0.28^c^	12.46 ± 0.08^de^
LC_50_ Eb + LC_50_ SS4	30.03 ± 0.23^e^	22.88 ± 0.34^g^	18.49 ± 0.34^e^	13.46 ± 0.38^ef^
LC_50_ Eb + LC_50_ EN4	43.73 ± 0.39^g^	28.33 ± 0.29^i^	25.40 ± 0.69^g^	18.03 ± 0.56^h^
LC_50_ Eb + LC_50_ M9	34.13 ± 0.32^f^	24.76 ± 0.03^h^	19.96 ± 0.29^f^	15.50 ± 0.35^g^
F_(9, 20)_—value	572.04**	2124.0**	1098.0**	346.87**

Figures are Mean ± Standard Error. Means followed by different superscript letters within a column are significantly different. Tukey’s test, p ≤ 0.05, **Significant at 1%

## Discussion

Bacterial isolates, *Shewanella* sp. (SS4), *Thauera* sp. (M9) and *Pseudomonas* sp. (EN4) were found to exhibit pathogenicity against *S. litura* in our previous experiments^23,24^. *Shewanella inventionis* HE_3_ and a number of *Pseudomonas* species such as *Pseudomonas chlororaphis*, *Pseudomonas taiwanensis*, *Pseudomonas fluorescens, Pseudomonas entomophila*, *Pseudomonas putida*, and *Pseudomonas paralactis* have been documented for insecticidal properties against many insect pests^25–29^. Pathogenicity of *Shewanella* sp. (SS4), *Thauera* sp. (M9) and *Pseudomonas* sp. (EN4) may be attributed to various hydrolytic enzymes viz. catalases, proteases, chitinases, lipases, oxidases and phospholipases which have been reported to be produced by these bacterial isolates^30–32^. All these bacterial isolates were found to be compatible with chlorantraniliprole and emamectin benzoate. Different combination treatments carried out in the present studies induced higher larval mortality in *S. litura* than individual bacterial or insecticide treatments.

Our findings indicated that even single application of insecticides followed by bacterial treatment increased the larval mortality in an additive manner. However, more than one application of insecticide alternating with bacteria enhanced the mortality and the interactions of bacteria and insecticides turned out to be synergistic. Among the two insecticides, bacterial cultures combined with chlorantraniliprole were found to be more effective showing mostly the synergistic effects even at low concentration (LC_20_) of insecticide. The increased mortality due to the additive effect as confirmed by Chi-square test, indicated that mortality observed in the combination treatments was caused by independent action of both bacterial isolates and insecticides whereas synergistic interaction demonstrated a significant interaction between two treatments^33^.

The combined use of bacteria with insecticides causes high mortality in pests, because chemical insecticides may act as stressor, weakening the immune response and increasing the susceptibility of insect to bacterial pathogens^34,35^. The anthranilic diamide, chlorantraniliprole is a new-generation insecticide and effective against lepidopteran insects that activate the ryanodine-sensitive intracellular calcium release channels (ryanodine receptor). The release of internal calcium stores leads to Ca^2+^ depletion, feeding cessation, lethargy, muscle paralysis and finally insect death^36,37^. Emamectin benzoate is a semi-synthetic derivative of abamectin that act as chloride channel activator, decreasing the excitability of neurons of lepidopterans and dipterans^38^. The insect larvae stop feeding immediately following exposure, become irreversibly paralyzed, and die within 3–4 days^16^.

Our results on comet assay indicated that combination treatments further enhanced DNA damage in hemocytes of *S. litura* as a significant increase was detected in all the comet parameters. It suggests that combined treatments cause more stress which further enhance pathogenicity and mortality in *S. litura* larvae. The studies are in line with the previous reports indicating genotoxicity due to insecticide exposure to various insects^39–41^. Microbial control agents have also been reported to cause genotoxicity, although very few reports are there^42,43^. Different pesticides such as delmithrin, endosulfan, malathion, cypermethrin, paraquat and λ–cyhalothrin etc. have been reported to increase the activity of oxidative stress enzyme^44–47^. This oxidative stress lead to production of reactive oxygen species (ROS) that disrupts the cellular redox balance causing lipid and protein oxidation as well as DNA damage^48^. The formation or lengthening of a tail is an indicator of the apoptosis process, as cells undergoing apoptosis exhibit nuclear fragmentation/disintegration in the form of DNA tails^49^. As the hemocytes play a vital role in providing defensive functions, thus direct effect of insecticidal and bacterial treatment may affect the cellular immune response by changing the viability and number of hemocytes, causing stress and making the insect more vulnerable to pathogens as well as suppressing the growth and developmental process.

Recent studies by Uma et al.^50^, documented sub-additive and synergistic effects of combination treatments of *B. thuringiensis* and chlorantraniliprole against *Spodoptera frugiperda* (J.E. Smith) larvae. An additive effect was observed between EPN species with LC_25_ and LC_50_ of emamectin benzoate on third instar larvae of cabbage white butterfy, *Pieris rapae* (Linneaus) after 3 days post-treatment^51^. Similar interactions were documented by other workers between entomopathogenic fungus and *Bt* against various insect pests^52–54^. Contrary to these studies, Amizadeh et al.^35^ reported antagonistic effect of *Bt* and insecticides when *Bt* was applied immediately after the applications of chemicals against tomato leafminer *Tuta absoluta* (Meyrick). Morales-Rodriguez and Peck^55^ also observed similar effect between *Bt* and neonicotinoid insecticides, imidacloprid and clothianidin against *Amphimallon majale* (Razoumowsky) and *Popillia japonica* (Newman), respectively. This antagonist effect may be due to incompatible nature of entomopathogenic fungi with chemical insecticides due to lower germination rate, decreased production of enzymes necessary for penetration of the insect’s cuticle, and poor mycelium growth ratio^56^. However, none of the associations were antagonistic in the present study rather these were additive and synergistic. Thus, use of biocontrol agents in combination with insecticides would not only increase the efficacy of biocontrol agents but also help to decrease the number of insecticide applications and thus help to reduce the load of chemical insecticides on environment. This strategy would ultimately result in improved pest management by natural enemies and finally delays the emergence of insecticide resistance.

## Materials and methods

### Rearing of insects

To establish the culture of *S. litura,* egg masses and larvae were collected from cauliflower and cabbage fields in and around Amritsar (Punjab), India. Mass rearing was carried out in the laboratory as per the protocol of Thakur et al.^57^ Larval rearing was carried out on fresh *Ricinus communis* leaves (Accession/Voucher number: 7590, identified from Department of Botanical and Environmental Sciences, Guru Nanak Dev University, Amritsar (Punjab), India) at controlled temperature of 25 ± 2^ο^C and 65 ± 5% humidity conditions respectively. The pupae were shifted to pupation jars (15 cm × 15 cm) having moist and sterilized sand. To facilitate egg-laying process, the adults were transferred to oviposition jars (15 cm × 15 cm) lined with filter paper. The adults were fed on honey solution (1 part honey to 4 parts water) soaked on a cotton swab that had been refreshed daily. The culture of *S. litura* was raised for three generations in the laboratory before employing for experiments.

### Bacterial cultures and preparation of bacterial stocks

Three bacterial isolates viz. *Shewanella* sp. (SS4), *Thauera sp.* (M9) and *Pseudomonas* sp*.* (EN4) (showing 98–99% nucleotide identity with *Shewanella xiamenensis* (GenBank accession number MZ268604), *Pseudomonas citronellolis* strain NBRC 103043 (NR114194) (GenBank accession no MW678603) and *Thauera humireducens* SgZ-1 (GenBank accession number MK619795)^58–60^, were procured from Department of Microbiology, Guru Nanak Dev University, Amritsar (Punjab), India, were found to exhibit insecticidal activity in our previous experiments^23,24^. These cultures were maintained on Luria Bertani (LB) plates. The bacteria were inoculated in LB broth and incubated at room temperature for 48 h at 30 °C. After centrifugation, the pellet was suspended in 1 ml phosphate buffer solution (PBS) (pH 7.0). The bacterial density was then optimized at optical density (OD_600_) at LC_50_ values for SS4, M9 and EN4.

### Insecticide formulation

The formulations of chlorantraniliprole (18.5% SC) and emamectin benzoate (5% SG) were purchased from FMC India Private Limited and Sinochem India Co. Private Limited respectively.

### Estimation of sub-lethal concentrations of insecticides

Preliminary bioassays with chlorantraniliprole and emamectin benzoate were performed to estimate the lethal concentrations killing second instar larvae of *S. litura*. A total of 50 larvae were used for each of the above mentioned five concentrations of both the insecticides. The stock solution (10 ppm) of each insecticide was prepared in 1000 ml distilled water and then serially diluted to prepare the five concentrations (0.0001 ppm, 0.001 ppm, 0.01 ppm, 0.1 ppm, and 1 ppm). Leaf dip method was adopted to conduct the experiment as per protocol of Sharma et al.^61^. The leaves treated with distilled water served as control. A single leaf disc (about 10 cm^2^) dipped in each insecticide concentration was air dried, cut into small pieces and placed in rearing tube containing *S. litura* larva. Only one larva was kept in each rearing tube. Each insecticide concentration was repeated five times (10 larvae per replication). The leaves were changed after 48 h of treatment. The experiments were conducted at constant temperature and humidity conditions of 25 ± 2 °C and 65 ± 5% respectively. Larval mortality was recorded after 24, 48 and 72 h of larval exposure to insecticide. LC_20_ and LC_50_ values were calculated after 72 h of treatment by Probit analysis. Larvae were considered dead when no movement of appendages was seen upon touching with a brush.

### Compatibility of bacteria and insecticide

To determine the compatibility of bacterial cultures with chlorantraniliprole, shake flask and plate assays were conducted. In the shake flask assay, three different concentrations of the insecticide (0.016 ppm, 0.1 ppm and 1.0 ppm) were added to 100 ml Luria Bertani broth in 250 ml flasks. The culture medium without insecticide served as control. A single colony of each bacterial isolate was inoculated into each treatment and control LB broth and incubated for 48 h at 30 °C and 180 rpm. Bacterial cultures were then centrifuged at 10,000 rpm and 4 °C for 10 min to observe the bacterial pellet growth. In the plate assay, the bacterial suspension of each culture (100 µL) was layered over LB plates with the help of a spreader. Then wells were made in four quadrants of plate. PBS was added to one of the well that served as control and three different concentrations of chlorantraniliprole (0.016 ppm, 0.1 ppm and 1.0 ppm) were added to the other three wells and left to dry for overnight. These plates were then incubated for 48 h at 30 °C to check the growth of bacterial cell suspensions. Similar procedure was followed for checking the compatibility of bacteria with emamectin benzoate. The compatibility of insecticide against bacterial cultures was demonstrated by diameter of clear or halo zones around the wells in comparison to the control well^62^.

### Effect of different concentrations of insecticide and bacteria against *S. litura*

For the individual treatments, the larvae were fed on sterile castor leaves treated with LC_50_ and LC_20_ concentrations of chlorantraniliprole and emamectin benzoate. The insecticide solutions were made in distilled water and experiment was carried out as mentioned above using leaf disc method. The leaves were changed after every 48 h till pupation. The larval mortality was observed on alternate days. Similarly for bacterial treatment, the larvae were fed on leaves treated with LC_50_ concentrations of bacterial cell suspensions (1.59 × 10^9^, 1.21 × 10^9^ and 1.67 × 10^9^ cfu/ml for SS4, M9 and EN4 respectively). The bacterial cell suspensions were prepared in PBS and the experimental procedure was same as used for insecticide treatment. All the insecticide and bacterial treatments were replicated five times (10 larvae per replication).

### Combined effects of bacteria and insecticide against *S. litura*

To evaluate the combined effect of insecticide and bacteria, the following combinations were taken:

**Table:** 

S. No	Treatments with chlorantraniliprole (Cg)	Treatments with emamectin benzoate (Eb)
1	LC_20_ Cg	LC_20_ Eb
2	LC_50_ Cg	LC_50_ Eb
3	LC_50_ SS4	LC_50_ SS4
4	LC_50_ EN4	LC_50_ EN4
5	LC_50_ M9	LC_50_ M9
6	LC_20_ Cg + LC_50_ SS4	LC_20_ Eb + LC_50_ SS4
7	LC_20_ Cg + LC_50_ EN4	LC_20_ Eb + LC_50_ EN4
8	LC_20_ Cg + LC_50_ M9	LC_20_ Eb + LC_50_ M9
9	LC_50_ Cg + LC_50_ SS4	LC_50_ Eb + LC_50_ SS4
10	LC_50_ Cg + LC_50_ EN4	LC_50_ Eb + LC_50_ EN4
11	LC_50_ Cg + LC_50_ M9	LC_50_ Eb + LC_50_ M9

Three different experiments were conducted on the basis of number of insecticide treatments given to the larvae. Second instar larvae of *S. litura* were selected and experiments were conducted using the leaf dip method. The experiments were replicated five times with ten larvae per replicate and laboratory conditions were maintained at 25 ± 2 °C temperature and 65 ± 5% relative humidity.

In the first experiment, larvae were treated with LC_50_ concentration of insecticide on the first day of experiment and 24 h post insecticide application, the larvae were treated with LC_50_ concentrations of bacterial cell suspensions (SS4, M9 and EN4). After that, only bacterial treatment was given on alternate days till pupation. Therefore, there was only one insecticide treatment.

In the second experiment, there were three insecticide treatments with alternate bacterial treatments. The first treatment with LC_50_ concentration of insecticide was followed by bacterial treatment after 24 h. Then second and third insecticidal treatments were given after 48 and 96 h with bacterial treatment in between at 72 h. After that the larvae were only treated with bacterial cell suspension on alternate days till pupation.

In the third experiment, there were six combination treatments that include two sub lethal concentrations of the insecticide i.e., LC_20_ and LC_50_. One set of larvae was fed on LC_20_ and the other set on LC_50_ concentration of insecticide. Insecticidal and bacterial treatments were given alternatively after every 24 h till pupation.

### Comet assay

Comet assay was done in alkaline conditions, using the protocol of Singh et al.^63^ with slight modifications. For the individual chlorantraniliprole treatments, the third-instar *S. litura* larvae were fed on LC_20_ concentration for 96 h. In the combination treatments of chlorantraniliprole, the larvae were fed on LC_20_ concentration of insecticide in alternation with LC_50_ concentrations of bacterial cell suspension (SS4, M9 and EN4) for 96 h. Similar procedure was followed for LC_50_ concentrations of chlorantraniliprole alone and in combination with bacterial isolates. Likewise the comet assay was also performed with emamectin benzoate and bacterial isolates. The prolegs of third instar larvae were shrugged off and hemolymph (from ten larvae per treatment) was collected in eppendorf tubes containing phosphate buffer. The slides were coated with 1% normal melting point agarose (NMPA) and hemocytes were layered on coated slides and kept in a refrigerator at 4 °C to settle down. The slides were then immersed in the lysing solution (2.5 M NaCl, 100 mM EDTA, 0.25 M Tris aminomethane, 0.25 M NaOH, 1% Triton X-100, 10% DMSO, double distilled water, pH 10.0), which was kept overnight in the refrigerator. Electrophoresis was performed using an electrophoretic unit (25 V; 300 mA) containing electrophoretic buffer (1 mM EDTA, 300 mM NaOH, double distilled water, pH > 13) for 20 min. The slides were neutralized for 15 min in a neutralization buffer (0.4 M Tris amino methane, double distilled water, pH 7.5). The slides were stained with 50 g/ml ethidium bromide and then dried. These were examined under a Nikon fluorescence microscope. Three replicates were used for each treatment. Using Casplab, the tail length, the Olive Tail Moment and the percentage of tail DNA were computed.

### Statistical analysis

One-way analysis of variance (ANOVA) was used to analyze differences in mortality data means with Tukey's test at p ≤ 0.05. The statistical analysis was carried out using SPSS software for windows version 16.0 (SPSS Inc, Chicago). Probit analysis was used to calculate the lower and median lethal values. A chi square test was used to determine whether the insecticide and bacterial samples had antagonistic, additive, or synergistic effect^64^. The formula ME = MB + MI (1 − MB/100) was used to determine the expected mortality value for bacterial-insecticidal interactions, where MB and MI stand for the observed mortality percentage brought on by the entomopathogenic bacteria and insecticide alone, respectively. The results of the χ^2^ test were compared with the χ^2^ table value for 1 degree of freedom using the formula χ^2^ = (MBI − ME)^2^/ME, where MBI is the observed mortality of the combination treatment. If the predicted value of χ^2^ is greater than the value in the table, a synergistic or antagonistic action between the two agents was found while if the tabular value is more than the χ^2^ value, an additive interaction was noticed. A significant interaction was observed to be synergistic if the difference between MBI and ME was positive; conversely, if it was negative, the interaction was deemed to be antagonistic. The comet parameters were replicated three times. The mean ± SE of all the values was used to represent them. One way analysis of variance (ANOVA) with Tukey's test at p ≤ 0.05 was used to compare differences in means.

### Ethical approval

The article does not contain any studies with human participants or vertebrate animals. No approval of research ethics committees was required to accomplish the goals of this study because experimental work was conducted with an unregulated invertebrate species.

## Conclusion

Combined applications of bacterial isolates with modest quantities of chlorantraniliprole and emamectin benzoate can effectively manage the lepidopteran pest *S. litura*. These combinations resulted in additive and synergistic effects, covering gaps of efficacy and improving the reliability of single agent treatments. More bacterial isolates should be investigated for their compatibility with insecticide against insect pests of agricultural importance. There is need to investigate the molecular interactions to ensure their best implementation into efficient, secure, and long-lasting IPM systems and verification in field circumstances.

## Data Availability

All the data generated and analyzed are included in this article.

## References

[1] Chattopadhyay N, Balasubramaniam R, Attri SD, Ray K, John G, Khedikar S, Karmakar C (2019). Forewarning of incidence of *Spodoptera litura* (Tobacco caterpillar) in soybean and cotton using statistical and synoptic approach. J. Agrimet..

[2] Fand BB, Sul NT, Bal SK, Minhas PS (2015). Temperature impacts the development and survival of common cutworm (*Spodoptera litura*): Simulation and visualization of potential population growth in India under warmer temperatures through life cycle modelling and spatial mapping. PLoS ONE.

[3] Ahmad M, Ghaffar A, Rafiq M (2013). Host plants of leaf worm, *Spodoptera litura* (Fabricius) (Lepidoptera: Noctuidae) in Pakistan. Asian J. Agric. Biol..

[4] Abbas N, Shad SA, Razaq M (2012). Fitness cost, cross resistance and realized heritability of resistance to imidacloprid in *Spodoptera litura* (Lepidoptera: Noctuidae). Pestic. Biochem. Physiol..

[5] Shi L, Shi Y, Zhang Y, Liao X (2019). A systemic study of indoxacarb resistance in *Spodoptera litura* revealed complex expression profiles and regulatory mechanism. Sci. Rep..

[6] Kranthi KR, Jadhav DR, Wanjari RR, Ali SS, Russell DA (2001). Carbamate and organophosphate resistance in cotton pests in India. Bull. Entomol. Res..

[7] Mishra J, Dutta V, Arora NK (2020). Biopesticides in India: Technology and sustainability linkages. 3 Biotech.

[8] Bravo A, Likitvivatanavong S, Gill SS, Soberón M (2011). *Bacillus thuringiensis*: A story of a successful bioinsecticide. Insect Biochem. Mol. Biol..

[9] Castagnola A, Stock SP (2014). Common virulence factors and tissue targets of entomopathogenic bacteria for biological control of lepidopteran pests. Insects.

[10] Ruiu L (2015). Insect pathogenic bacteria in integrated pest management. Insects.

[11] Lacey LA, Grzywacz D, Shapiro-Ilan DI, Frutos R, Brownbridge M, Goettel MS (2015). Insect pathogens as biological control agents: Back to the future. J. Invertebr. Pathol..

[12] Liu FH, Lin XL, Kang ZW, Tian HG, Liu TX (2019). Isolation and characterization of *Pseudomonas cedrina* infecting *Plutella xylostella* (Lepidoptera: Plutellidae). Arch. Insect Biochem. Physiol..

[13] El-Ashry RM, Ramadan MM (2021). In Vitro Compatibility and Combined Efficacy of Entomopathogenic Nematodes with Abamectin and Imidacloprid Against the White Grub, *Pentodon*
*bispinosus* Kust. Egypt. Acad. J. Biol. Sci. F..

[14] Robertson JL, Jones MM, Olguin E, Alberts B (2017). Bioassays with Arthropods.

[15] Singh AK, Singh A, Joshi P (2016). Combined application of chitinolytic bacterium *Paenibacillus* sp. D1 with low doses of chemical pesticides for better control of *Helicoverpa*
*armigera*. Int. J. Pest Mang..

[16] Grafton-Cardwell E, Godfrey L, Chaney W, Bentley W (2005). Various novel insecticides are less toxic to humans, more specific to key pests. Calif. Agric..

[17] Ishaaya I, Horowitz AR, Ishaaya I, Degheele D (1998). Insecticides with novel modes of action: An overview. Insecticides with Novel Modes of Action, Mechanism and Application.

[18] El-Sheikh ESA (2015). Comparative toxicity and sublethal effects of emamectin benzoate, lufenuron and spinosad on *Spodoptera*
*littoralis* Boisd. (Lepidoptera: Noctuidae). Crop Prot..

[19] Konecka E, Kaznowski A, Grzesiek W, Nowicki P, Czarniewska E, Baranek J (2020). Synergistic interaction between carvacrol and *Bacillus thuringiensis* crystalline proteins against *Cydia pomonella* and *Spodoptera exigua*. Biocontrol.

[20] Niu H, Wang N, Liu B, Xiao L, Wang L, Guo H (2018). Synergistic and additive interactions of *Serratia marcescens* S-JS1 to the chemical insecticides for controlling *Nilaparvata lugens* (Hemiptera: Delphacidae). J. Econ. Entomol..

[21] Paula AR, Carolino AT, Paula CO, Samuels RI (2011). The combination of the entomopathogenic fungus *Metarhizium anisopliae* with the insecticide Imidacloprid increases virulence against the dengue vector *Aedes aegypti* (Diptera: Culicidae). Parasites Vectors.

[22] Bitsadze N, Jaronski S, Khasdan V, Abashidze E, Abashidze M, Latchininsky A, Samadashvili D, Sokhadze I, Rippa M, Ishaaya I, Horowitz AR (2013). Joint action of *Beauveria bassiana* and the insect growth regulators diflubenzuron and novaluron, on the migratory locust, *Locusta migratoria*. J. Pest Sci..

[23] Sarkhandia S, Devi M, Sharma G, Mahajan R, Chadha P, Saini HS, Kaur S (2023). Larvicidal, growth inhibitory and biochemical effects of soil bacterium, *Pseudomonas* sp. EN4 against *Spodoptera*
*litura* (Fab.) (Lepidoptera: Noctuidae). BMC Microbiol..

[24] Sarkhandia S, Devi S, Sharma G, Kumar M, Koundal S, Chadha P, Saini HS, Kaur S (2023). Insecticidal, genotoxic and biochemical effects of *Shewanella *sp. (SS4) and *Thauera* sp. (M9) on Spodoptera litura. J. Appl. Entomol..

[25] Ruffner B, Péchy-Tarr M, Ryfel F, Hoegger P, Obrist C, Rindlisbacher A, Keel C, Maurhofer M (2013). Oral insecticidal activity of plant-associated pseudomonads. Environ. Microbiol..

[26] Chen WJ, Hsieh FC, Hsu FC, Tasy YF, Liu JR, Shih MC (2014). Characterization of an insecticidal toxin and pathogenicity of *Pseudomonas taiwanensis* against insects. PLoS Pathog..

[27] Dieppois G, Opota O, Lalucat J, Lemaitre B (2015). *Pseudomonas entomophila*: A versatile bacterium with entomopathogenic properties. Pseudomonas.

[28] Laribi-Habchi H, Bouacem K, Allala F, Jabeur F, Selama O, Mechri S, Yahiaoui M, Bouanane-Darenfed A, Jaouadi B (2020). Characterization of chitinase from *Shewanella inventionis* HE3 with bio-insecticidal effect against granary weevil, *Sitophilus granarius* Linnaeus (Coleoptera: Curculionidae). Process Biochem..

[29] Devi S, Saini HS, Kaur S (2022). Insecticidal and growth inhibitory activity of gut microbes isolated from adults of *Spodoptera*
*litura* (Fab.). BMC Microbiol..

[30] Tarhriz V, Mohammadzadeh F, Hejazi MS, Nematzadeh G, Rahimi E (2011). Isolation and characterization of some aquatic bacteria from Qurugol Lake in Azerbaijan under aerobic conditions. Adv. Environ. Biol..

[31] Ng IS, Xu F, Zhang X, Ye C (2015). Enzymatic exploration of catalase from a nanoparticle producing and biodecolorizing algae *Shewanella xiamenensis* BC01. Bioresour. Technol..

[32] Loper JE, Henkels MD, Rangel LI, Olcott MH, Walker FL, Bond KL, Kidarsa TA, Hesse CN, Sneh B, Stockwell VO, Taylor BJ (2016). Rhizoxin analogs, orfamide A and chitinase production contribute to the toxicity of *Pseudomonas protegens* strain Pf-5 to *Drosophila melanogaster*. Environ. Microbiol..

[33] Wakil W, Ghazanfar MU, Riasat T, Qayyum MA, Ahmed S, Yasin M (2013). Effects of interactions among *Metarhizium anisopliae*, *Bacillus thuringiensis* and chlorantraniliprole on the mortality and pupation of six geographically distinct *Helicoverpa armigera* field populations. Phytoparasitica.

[34] Vallet-Gely I, Lemaitre B, Boccard F (2008). Bacterial strategies to overcome insect defences. Nat. Rev. Microbiol..

[35] Amizadeh M, Hejazi MJ, Niknam G, Arzanlou M (2015). Compatibility and interaction between *Bacillus thuringiensis* and certain insecticides: Perspective in management of *Tuta absoluta* (Lepidoptera:Gelechiidae). Biocontrol Sci. Technol..

[36] Caboni P, Sarais G, Angioni A, Vargiu S, Pagnozzi D, Cabras P, Casida JE (2008). Liquid chromatography−tandem mass spectrometric ion-switching determination of chlorantraniliprole and flubendiamide in fruits and vegetables. J. Agric. Food Chem. J..

[37] Lavtizar V, Helmus R, Kools SA, Dolenc D, van Gestel CA, Trebsse P, Waaijers SL (2015). Daphnid life cycle responses to the insecticide chlorantraniliprole and its transformation products. Environ. Sci. Technol..

[38] Stanley J, Chandrasekaran S, Regupathy A, Sheeba-Jasmine R (2006). Base line toxicity of emamectin and spinosad to *Spodoptera litura*. Ann. Plant Protect. Sci..

[39] Morrissey CA, Mineau P, Devries JH, Sanchez-Bayo F, Liess M, Cavallaro MC, Liber K (2015). Neonicotinoid contamination of global surface waters and associated risk to aquatic invertebrates: A Review. Environ. Int..

[40] Wu X, Zhang L, Yang C, Zong M, Huang Q, Tao L (2016). Detection on emamectin benzoate-induced apoptosis and DNA damage in *Spodoptera frugiperda* Sf-9 cell line. Pestic. Biochem. Physiol..

[41] Saleh M, Ezz-din D, Al-Masri A (2021). In vitro genotoxicity study of the lambda-cyhalothrin insecticide on Sf9 insect cells line using Comet assay. Jordan J. Biol. Sci..

[42] Oberholster PJ, Mthethwa B, Botha AM (2009). Development of a rapid and sensitive battery of bioassays for risk assessment of cyanobacterial microcystin-LR in drinking water of rural water treatment plants, South Africa. Afr. J. Biotechnol..

[43] Kaur M, Chadha P, Kaur S, Kaur A, Kaur R, Yadav AK, Kaur R (2018). *Schizophyllum commune* induced genotoxic and cytotoxic effects in *Spodoptera litura*. Sci. Rep..

[44] El-Demerdash FM (2007). Lambdacyhalothrin-induced changes in oxidative stress biomarkers in rabbit erythrocytes and alleviation effect of some antioxidants. Toxicol. In Vitro.

[45] Fetoui H, Makni M, Garoui M, Zeghal N (2010). Toxic effects of lambda-cyhalothrin, a synthetic pyrethroid pesticide, on the rat kidney: Involvement of oxidative stress and protective role of ascorbic acid. Exp. Toxicol. Pathol..

[46] Madkour NK (2012). Protective effect of curcumin on oxidative stress and DNA fragmentation against lambda cyhalothrin-induced liver damage in rats. J. Appl. Pharm. Sci..

[47] Ullah S, Begum M, Dhama K, Ahmad S, Hassan S, Alam I (2016). Malathion induced DNA damage in freshwater fish, *Labeo rohita* (Hamilton, 1822) using alkaline single cell gel electrophoresis. Asian J. Anim. Vet. Adv..

[48] Halliwell B, Gutteridge JMC (2015). Free Radicals in Biology and Medicine.

[49] Porichha SK, Sarangi PK, Prasad R (1998). Genotoxic effect of chlorpyrifos in *Channa punctatus*. Pres. Cytol. Genet..

[50] Uma D, Varanavasiappan S, Geetha S, Sathiah N, Muthuswami M, Gowtham V (2022). Assessing the Single and Combined Toxicity of Chlorantraniliprole with *Bacillus thuringiensis* against Maize Fall Armyworm *Spodoptera frugiperda* (JE Smith) (Lepidoptera: Noctuidae) under Laboratory Conditions. Int. J. Plant Soil Sci..

[51] Aioub AA, El-Ashry RM, Hashem AS, Elesawy AE, Elsobki AE (2021). Compatibility of entomopathogenic nematodes with insecticides against the cabbage white butterfly, *Pieris*
*rapae* L. (Lepidoptera: Pieridae). Egypt. J. Biol. Pest Control.

[52] Ansari MA, Tirry L, Moens M (2004). Interaction between *Metarhizium anisopliae* CLO 53 and entomopathogenic nematodes for the control of *Hoplia philanthus*. Biol. Control.

[53] Kryukov VY, Khodyrev VP, Yaroslavtseva ON, Kamenova AS, Duisembekov BA, Glupov VV (2009). Synergistic action of entomopathogenic hyphomycetes and the bacteria *Bacillus*
*thuringiensis* ssp. morrisoni in the infection of Colorado potato beetle *Leptinotarsa*
*decemlineata*. Appl. Biochem. Microbiol..

[54] Gao Y, Oppert B, Lord JC, Liu C, Lei Z (2012). *Bacillus thuringiensis* Cry3Aa toxin increases the susceptibility of *Crioceris quatuordecimpunctata* to *Beauveria bassiana* infection. J. Invertebr. Pathol..

[55] Morales-Rodriguez A, Peck DC (2009). Synergies between biological and neonicotinoid insecticides for the curative control of the white grubs *Amphimallon majale* and *Popillia japonica*. Biol. Control.

[56] Bednarek A, Popowska-Nowak E, Pezowicz E, Kamionek M (2004). Integrated methods in pest control: Effect of insecticides on entomopathogenic fungi [*Beauveria*
*bassiana* [Bals] Vuill, *B*
*brongniartii* [Sacc]] and nematodes [*Heterorhabditis*
*megidis* Poinar, Jackson, Klein, *Steinernema*
*feltiae* Filipjev, *S*.*Glaseri* Steiner]. Polish J. Ecol..

[57] Thakur A, Dhammi P, Saini HS, Kaur S (2015). Pathogenicity of bacteria isolated from gut of *Spodoptera litura* (Lepidoptera: Noctuidae) and fitness costs of insect associated with consumption of bacteria. J. Invertebr. Pathol..

[58] Koundal S, Sharma K, Dhammi P, Chadha P, Saini HS (2023). Development and operation of immobilized cell plug flow bioreactor (PFR) for treatment of textile industry effluent and evaluation of its working efficiency. Environ. Sci. Pollution Res..

[59] Mahajan R, Sharma G, Koundal S, Chadha P, Kumar S, Saini HS (2022). Co-metabolism of 4-bromophenol by *Pseudomonas* sp. EN-4 and toxicity evaluation of biotransformed samples. J. Environ. Chem. Eng..

[60] Kumar M, Mahajan R, Saini HS (2020). Evaluating metabolic potential of *Thauera* sp. M9 for the transformation of 4-chloroaniline (4-CA). Biocat. Agricult. Biotechnol..

[61] Sharma S, Kaur A, Kooner R (2019). Relative toxicity of newer insecticides against *Spodoptera litura* and *Pieris brassicae* infesting Cole crops in Punjab. Indian J. Horticult..

[62] Ahemad, M. & Khan, M. S. Effects of insecticides on plant-growth-promoting activities of phosphate solubilizing rhizobacterium *Klebsiella* sp. strain PS19. *Pestic. Biochem. Physiol.* **100**(1), 51–56. 10.1016/j.pestbp.2011.02.004 (2011).

[63] Singh NP, McCoy MT, Tice RR, Schneider EL (1988). A simple technique for quantitation of low levels of DNA damage in individual cells. Exp. Cell Res..

[64] Koppenhofer AM, Fuzy EM (2008). Early timing and new combinations to increase the efficacy of neonicotinoid–entomopathogenic nematode (Rhabditida: Heterorhabditidae) combinations against white grubs (Coleoptera: Scarabaeidae). Pest Manag. Sci..

